# Artificial intelligence-based recognition for variant pathogenicity of BRCA1 using AlphaFold2-predicted structures

**DOI:** 10.7150/thno.79362

**Published:** 2023-01-01

**Authors:** Chang Li, Lili Zhang, Zhongling Zhuo, Fei Su, Hexin Li, Siyuan Xu, Ye Liu, Zaifeng Zhang, Yibo Xie, Xue Yu, Liheng Bian, Fei Xiao

**Affiliations:** 1 Peking University Fifth School of Clinical Medicine, Beijing, China.; 2 Clinical Biobank, Beijing Hospital, National Center of Gerontology, National Health Commission, Institute of Geriatric Medicine, Chinese Academy of Medical Sciences, Beijing, China.; 3 Department of Clinical Laboratory, Peking University People's Hospital, Beijing, China.; 4 The Key Laboratory of Geriatrics, Beijing Institute of Geriatrics, Beijing Hospital, National Center of Gerontology, National Health Commission, Institute of Geriatric Medicine, Chinese Academy of Medical Sciences, Beijing, China.; 5 Information Center, Beijing Hospital, National Center of Gerontology, National Health Commission, Institute of Geriatric Medicine, Chinese Academy of Medical Sciences, Beijing, China.; 6 Department of Cardiology, Beijing Hospital, National Center of Gerontology, Institute of Geriatric Medicine, Chinese Academy of Medical Sciences, Beijing, China.; 7 Advanced Research Institute of Multidisciplinary Science, Beijing Institute of Technology, Beijing, China.

**Keywords:** artificial intelligence, gene variation, clinical interpretation, deep learning, tertiary protein structure

## Abstract

With the surge of the high-throughput sequencing technologies, many genetic variants have been identified in the past decade. The vast majority of these variants are defined as variants of uncertain significance (VUS), as their significance to the function or health of an organism is not known. It is urgently needed to develop intelligent models for the clinical interpretation of VUS. State-of-the-art artificial intelligence (AI)-based variant effect predictors only learn features from primary amino acid sequences, leaving out information about the most important three-dimensional structure that is more related to its function.

**Methods:** We proposed a deep convolutional neural network model named variant effect recognition network for BRCA1 (vERnet-B) to recognize the clinical pathogenicity of missense single-nucleotide variants in the BRCT domain of BRCA1. vERnet-B learned features associated with the pathogenicity from the tertiary protein structures of variants predicted by AlphaFold2.

**Results:** After performing a series of validation and analyses on vERnet-B, we discovered that it exhibited significant advances over previous works. Recognizing the phenotypic consequences of VUS is one of the most daunting challenges in genetic informatics; however, we achieved 85% accuracy in recognizing disease BRCA1 variants with an ideal balance of false-positive and true-positive detection rates. vERnet-B correctly recognized the pathogenicity of variant A1708E, which was poorly predicted by AlphaFold2 as previously described. The vERnet-B web server is freely available from URL: http://ai-lab.bjrz.org.cn/vERnet.

**Conclusions:** We applied protein tertiary structures to successfully recognize the pathogenic missense SNVs, which were difficult to be addressed by classical approaches based on sequences. Our work demonstrated that AlphaFold2-predicted structures were expected to be used for rich feature learning and revealed unique insights into the clinical interpretation of VUS in disease-related genes, using vERnet-B as a discovery tool.

## Introduction

Identifying the pathogenicity of human disease-associated gene variants, especially missense single-nucleotide variants (SNVs), is crucial for clinical decisions. For example, deleterious genetic mutations in BRCA1, a tumor suppressor gene, can impair protein function and raise the risk of multiple cancers 1-3. However, not all BRCA1 variants contribute to cancer risk. If the phenotypic consequences of an arbitrary genetic variant in BRCA1 can be determined, the prognosis can be improved after frequent screening, prophylactic surgery, and/or precision treatment 4-6. Although BRCA1 has been sequenced in millions of women, only a small proportion of BRCA1 variants had definite clinical interpretations. As of February 2022, most of the BRCA1 variants in ClinVar were classified as variants of uncertain significance (VUS) 7. Novel missense SNVs are typical VUS, and their roles in BRCA1 warrant further investigations. To address this challenge, various technologies that can assess the functional effects of mutations have emerged 8-10. Gregory et al. used saturation genome editing (SGE) to measure the functional consequences for all possible SNVs in some functionally critical domains of BRCA1 10, 11. Although the effects of 3893 SNVs have been characterized, only the sites affecting two functional domains of BRCA1 were explored, whereas the damaging mutations could occur anywhere in BRCA1 12. Furthermore, SGE can be quite expensive and time-consuming when applied to the whole BRCA1 gene. Some studies demonstrated that artificial intelligence (AI) also accelerated the clinical interpretation of VUS 13-16. Notably, these AI projects were performed mainly based on their primary sequences, and the reflection between their 3-dimensional structures of the variants and their biological functions, such as the RNA binding properties and protein stability, had not been established for the deep learning, due to the limited number of whole-protein structures stored in the Protein Data Bank (PDB) 17. In 2020, the AlphaFold2 project 18, 19, developed by Google's AI project Alpha and their British division DeepMind (https://deepmind.com), stood out in the challenging 14th Critical Assessment of protein Structure Prediction (CASP14), with outstanding >90% global distance test (GDT, an accuracy index). AlphaFold2 overcame the enormous economic and time demands in determining protein structures and enabled the efficient obtaining of more tertiary protein structures. Compared with the widespread technique, homology modeling (HM) 20, AlphaFold2 shows higher availability for several reasons: (i) there is no need for a close homolog solved experimentally; (ii) the whole-protein structure can be obtained; (iii) the AI algorithms provide unlimited potential for refining the structures 21. However, some authors indicated that AlphaFold2 seemed to fall short in predicting the structural effects of missense mutations as AlphaFold2 predicted similar structures for wild-type (WT) and missense mutant proteins 22. In contrast, our previous work found that constructing amino acid interaction networks (AANs) from AlphaFold2-predicted structures could provide more information to distinguish between WT and mutant proteins, thus helping to characterize the feature of pathogenicity.

Therefore, we developed a learning model for inferring protein function from their tertiary structures, which facilitates the functional classification of BRCA1 variants relying on their tertiary structures with deep learning. We collected missense SNVs with known pathogenicity in the BRCT domain of BRCA1 from ClinVar 7, BRCAExchange 23, and SGE function scores 10. AlphaFold2 was used to predict the tertiary protein structures of these variants, and then the corresponding AANs were constructed. Next, we employed Convolutional Neural Networks (CNNs) to train a model named vERnet-B, which could recognize the clinical pathogenicity of BRCA1 variants. The performances of vERnet-B were systematically validated with a large number of independent data. Overall, the application of vERnet-B to the clinical interpretation of BRCA1 variants provided a novel cost-effective and reliable functional annotation method of VUS. Instead of the labor-intensive wet-lab experiments, vERnet-B can overcome the obstacles that previous VUS functional prediction was limited to a few of extensively studied functional domains. Moreover, we successfully applied this method to another functional domain of BRCA1, RING. It provided a high thought-put tool to explore the much broader unknown domains of BRCA1 as well as other genes, allowing us to eliminate or reduce costly and time-consuming functional studies for inferring the pathogenicity of variants in the future.

## Results

### Recognizing pathogenicity from tertiary protein structures

**Figure 1:**
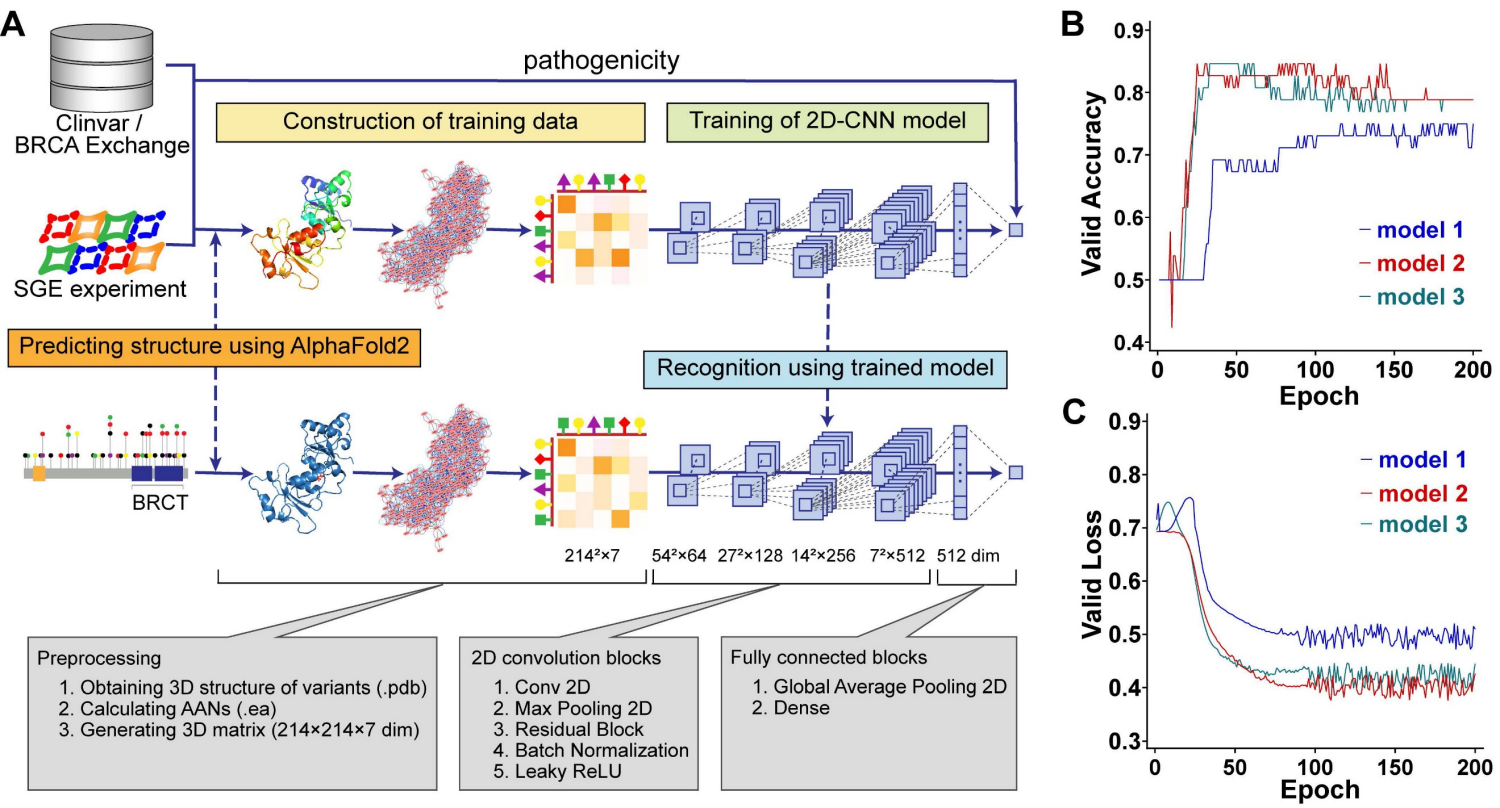
**|** Complete workflow of training a model for recognizing the clinical pathogenicity of missense SNVs in BRCA1 and using it on independent data. (A) Model training was carried out using BRCA1 variants and their pathogenicity class derived from ClinVar, BRCA Exchange, and SGE function scores. In the training-data construction stage, the matrices representing AANs were derived based on the tertiary structures predicted by AlphaFold2 (Method, see the sections “Construction of amino acid networks” and “Data preparation for AANs learning”). In the training stage, the 2D-CNN model called vERnet-B learned the relationship between the pathogenicity and tertiary structure of protein variants (Method, see the section “Construction of deep neural networks”). In the recognition stage, for variants that were not included in the training dataset, vERnet-B was used to recognize pathogenicity based on the input AANs matrices. In this study, 659 variants were used to train and validate the vERnet-B, and additional 484 variants were used for pathogenicity recognition using vERnet-B and further combining methods. (B) The accuracy validation of vERnet-B over training time, in units of epochs. The three colors of curves represented the three independent base classifiers trained using ResNet18. (C) The loss function validation of vERnet-B over training time, in units of epochs. The three colors of curves represented the three independent base classifiers trained using ResNet18.

Our method aimed to learn the pathogenic propensity of human missense SNVs in BRCA1 from the protein tertiary structures (Figure 1A). We used AlphaFold2 to obtain the PDB files representing the protein tertiary structures, which were used to generate AANs by the RINerator module 24, and then were transformed into 3D matrices that could be used by deep learning algorithms (Figure 1A See Construction of training data stage). Next, we trained two-dimensional convolutional neural network (2D-CNN) models to learn the relationship between the pathogenicity and tertiary structures of protein variants (Figure 1A see Training of 2D-CNN model stage). The final model vERnet-B consisted of three base classifiers, whose accuracy and loss function on the validation dataset converged well within 200 training epochs (Figure 1 B-C). In this study, the BRCT domain of BRCA1 is a protein fragment containing 214 amino acids. The 3D matrices (214 × 214 × 7) were extracted from their AANs (EA files) as input data for deep learning, and the 2D-CNN model was trained to recognize the pathogenicity of each variant. Finally, using the trained 2D-CNN model, we extracted the hidden pathogenicity information of BRCA1 missense SNVs occurring in the BRCT domain (Figure 1A see Recognition stage). In the recognition phase, only the amino acid substitution information of the variants was required.

**Figure 2:**
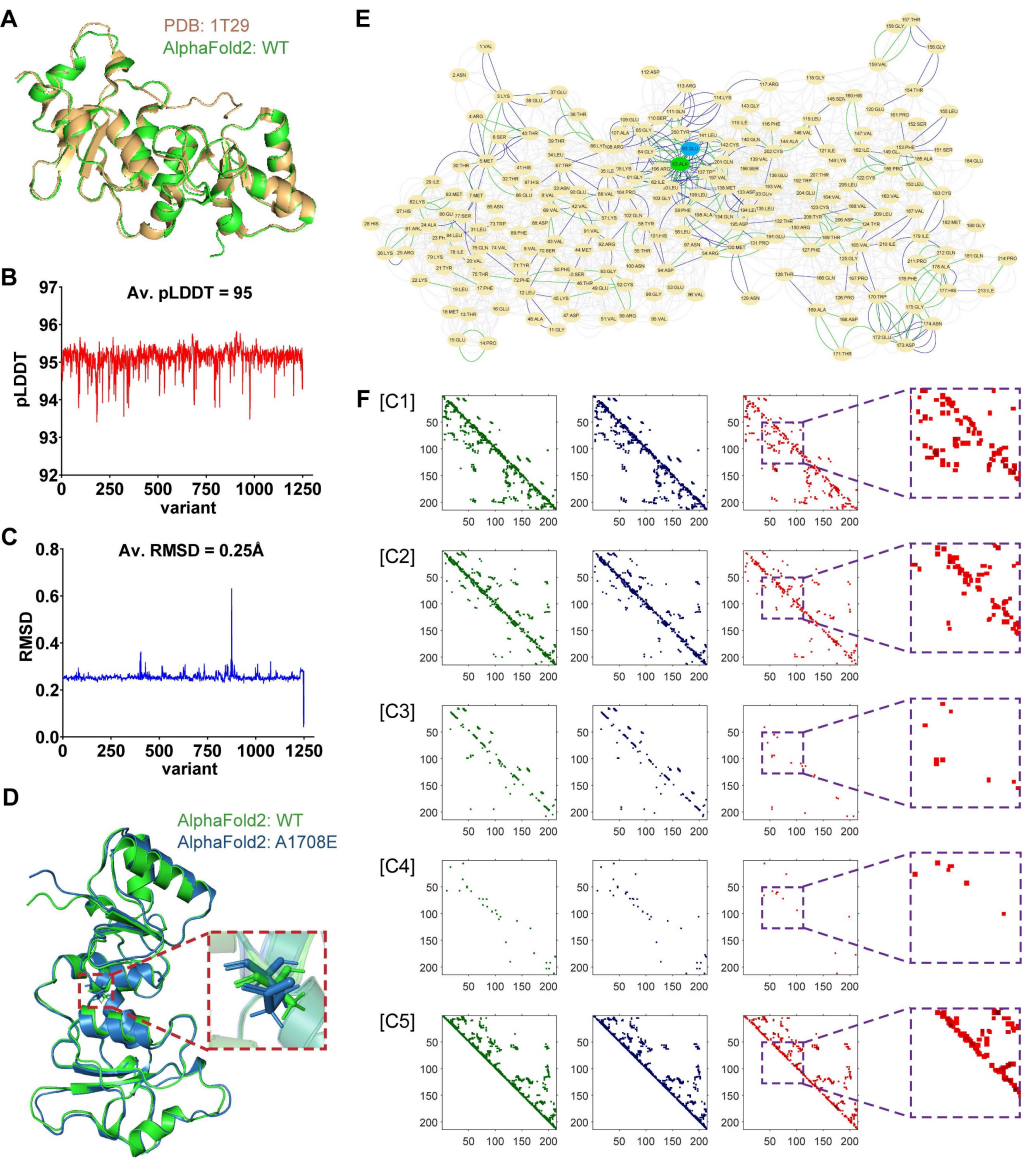
**|** Protein tertiary structure representation for training 2D-CNN model. (A) Alignment and overlay of PDB entry 1T29 (gold) and AlphaFold2-predicted WT (green) BRCT. A high degree of consistency between the two structures was shown, with an average Cα r.m.s.d. of only 0.632 Å. (B) The average value of mean pLDDT scores evaluated by AlphaFold2 for each variant structure. (C) The average Cα r.m.s.d. between each AlphaFold2-predicted variant structure and WT BRCT. (D) Alignment and overlay of AlphaFold2-predicted WT (green) and A1708E (blue) BRCT. Detail residues of Ala-1708 and Glu-1708 were highlighted. (E) Comparison and overlay of the AANs for WT (highlight the different edges in green) and A1708E (highlight the different edges in blue) BRCT. (F) Visualization of each channel in the input matrices for training, where the information of WT, A1708E, and the difference between them were respectively drawn in green, blue, and red. Channels 1 to 7 respectively stored the cnt (mc_mc and sc_sc), cnt(mc_sc), hbond (mc_mc and sc_sc), hbond (mc_sc), combi(all_all), ovl (mc_mc and sc_sc) and ovl (mc_sc). Channels 6 and 7 for both WT and A1708E BRCT were almost empty, thus we didn't display them here.

The currently available experimental techniques for determining protein structures include X-ray crystallography, nuclear magnetic resonance (NMR) spectroscopy, and cryo-electron microscopy (cryo-EM) 25-27. It is difficult to obtain enough protein structures of variants for deep learning from these experimental methods. In our current study, we used the open-source AlphaFold2 for protein tertiary structure prediction. The average root-mean-square deviation (r.m.s.d.) on backbone α-carbon (Cα) atoms was used to quantify the similarity between two protein tertiary structures. The mean score of the predicted local-distance difference test (pLDDT) was used to reflect the confidence of AlphaFold2-predicted structures. We first predicted the tertiary structure of the WT BRCT domain of BRCA1 using AlphaFold2, which was almost identical to the natural structure measured by X-ray diffraction in the PDB database (PDB entry: 1T29), with an average Cα r.m.s.d. of only 0.434 Å (Figure 2A). Next, we predicted the tertiary structures of 1143 missense SNVs in BRCT collected from three platforms, including Cinvar, BRCA Exchange, and SGE function scores. The average value of mean pLDDT scores for all these variant structures was 95 (Figure 2B), and the mean pLDDT score for the WT structure was 94.2, indicating that AlphaFold2 was highly reliable for the structure predictions of missense SNVs.

Since missense SNVs only cause the substitution of one amino acid, they usually have little effect on the coiling and folding of the protein structures, resulting in a mean value of average Cα r.m.s.d. of only 0.25Å when comparing all variants to WT (Figure 2C). Therefore, RINerator was applied to deduce additional biochemical information to construct the amino acid interaction network based on the atomic spatial position information in the PDB file 24. The AAN is an undirected weighted network with multiple edges that is capable of quantitatively representing multiple biochemical interactions between amino acid residues. The nodes represent the amino acid residues of the protein, whereas the links between them represent the non-covalent interactions including interatomic contact, hydrogen bond, overlap, and generic residue interaction. Figure 2D-F showed how protein tertiary structures can be predicted and represented during the preprocessing phase, using WT and the missense variant A1708E located in the BRCT domain as an example. In previous proteolytic degradation assays, A1708E was found to destabilize the BRCA1 C-terminal repeat 28, 29. It was also classified as a pathogenic mutation in ClinVar. However, AlphaFold2 predicted very similar 3D structures for WT and A1708E BRCT (Figure 2D), with an average Cα r.m.s.d. of only 0.632 Å 22. In contrast, the comparison of their AANs that were generated from the 3D structures (Figure 2E) showed that the substitution of one amino acid not only affected its neighboring residue interactions but also had a role in the global networks to some extent. After incorporating their multiple interactions into the 3D matrices (Figure 2F), we found that the most significant differences between WT and A1708E BRCT were presented in channels 1, 2, and 5, bearing the information of interatomic contact and overlap. In channels 3 and 4 that stored the information of hydrogen bonds between the main chains and side chains, there were some differences in the region near the variant site. We aligned the remaining nearly 1200 missense SNVs with WT, and found that all of them followed the above difference rule.

Finally, we leveraged the 3D matrices of AANs for learning the relationship between AANs and their pathogenicity. We collected 1143 missense SNVs in BRCT from three platforms including ClinVar, BRCA Exchange, and SGE function scores (see Supplementary Information), where the number of positive samples was less than 1/3 compared to the number of negative samples. To efficiently utilize this imbalanced dataset, we proposed an under-sampling schema using the EasyEnsemble 30. In this schema, we trained three CNN models as base classifiers, and the training dataset of each individual base classifier was obtained by repeatedly combining 260 positive samples with the same number of randomly sampled negative samples. Each base classifier was implemented by ResNet18 31 (see Methods for more information on CNN network structure and EasyEnsemble). The learned model of vERnet-B was obtained by integrating the results of three base classifiers. Here, instead of directly taking the results (0,1) of all three base classifiers for voting, a more reliable classification result was determined based on their probabilities.

### vERnet-B recognizes BRCA1 pathogenicity in an independent dataset

**Figure 3:**
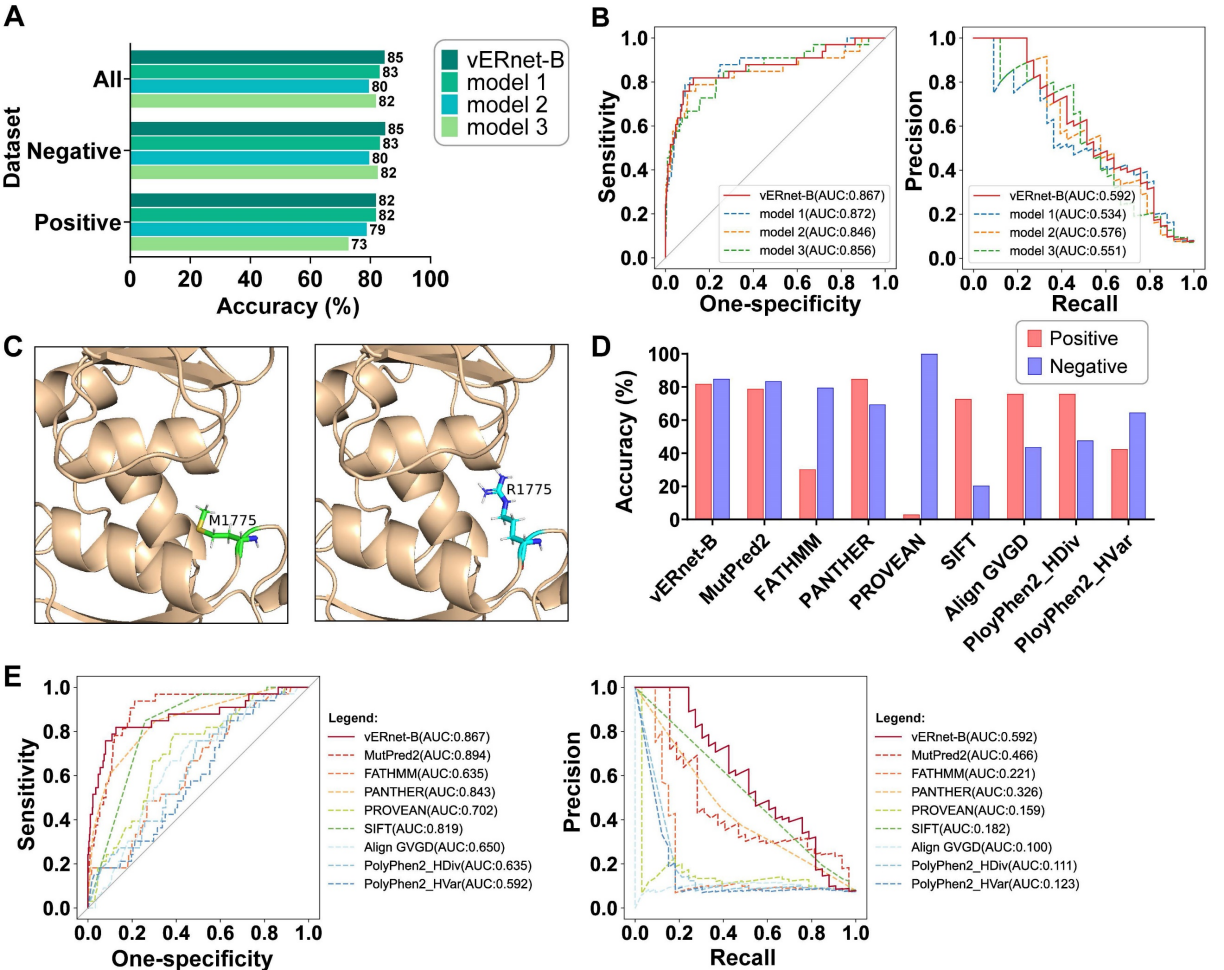
**|** Validation and evaluation of vERnet-B. (A) Testing accuracy in the positive, negative, and integrated testing datasets by vERnet-B and three base classifiers. (B) The AUC values of the ROC and PR of the recognized pathogenicity ranks (bigger ranks refer to stronger risks) for the 484 BRCT variants in the testing dataset were shown. The red solid curve represented the performance of the final model of vERnet-B, while the dashed curves represented the performance of the base classifiers. (C) The local structures proximal to the mutation site of WT (left) and M1775R (right) BRCT. The side chain of Arg-1775 has been extruded from the hydrophobic pocket while the Met-1775 was normally packed. (D) Performance comparison of vERnet-B to other eight computational variant effect predictors in the positive (red) and the negative (blue) testing datasets. (E) The AUC values of the ROC and PR of the recognized pathogenicity ranks (bigger ranks refer to stronger risks) for the 484 BRCT variants in the testing dataset were shown. The solid curve represented the performance of vERnet-B, while the dashed curves represented the performance of other eight computational variant effect predictors.

A series of validation was performed using a testing dataset containing a large number of independent missense SNVs in the BRCT domain of BRCA1 with supporting evidence for pathogenicity. In this and all of the following validation analyses, all variants sampled in the training dataset were removed from the testing dataset. Moreover, to provide a more robust assessment, synonymous SNVs that can cause the same amino acid substitutions were removed from the testing dataset, considering those synonymous SNVs may result in conflicting clinical interpretations. A total of 484 variants were included in the testing dataset, in which the 33 pathogenic variants were regarded as positive samples while the else 451 benign variants were regarded as negative samples. An accuracy of up to 85% for benign and pathogenic classification was yielded in the testing dataset (Figure 3A). In addition, the 82% and 85% accuracies were respectively achieved for recognizing the pathogenic and benign variants, demonstrating that vERnet-B truly learned the features related to pathogenicity, rather than the selection bias to positive or negative samples. Due to the imbalance in the number of negative and positive samples, we used two metrics, the area under the curve (AUC) of the Precision-Recall (PR) and Receiver Operating Characteristic (ROC) for evaluating the performance of vERnet-B. Strikingly, the AUC values of the ROC and the PR reached 0.87 and 0.59 respectively in the testing cohort for vERnet-B (Figure 3B). The vERnet-B prediction using the integrated results from EasyEnsemble had a superior accuracy compared to the three base classifiers in Figure 3A-B. Then we took a missense variant M1775R as an example to illustrate the accurate recognition capability of our method. In the previously reported proteolytic assays, the amino acid substitution from Met-1775 to Arg-1775 would extrude the mutated side chain from the protein hydrophobic core, which led to the conformational instability of BRCT-M1775R 32. As shown in Figure 3C, AlphaFold2 successfully predicted the same structural change as the proteolytic assay, and then vERnet-B also correctly recognized BRCT-M1775R as a pathogenic mutation with a high score of more than 99%.

We compared the performance of vERnet-B to other eight computational variant effect predictors using a testing dataset consisting of 484 missense SNV variants. These methods are independent of the ClinVar labeling process, as well as, in some cases, being used extensively to assist in defining clinical interpretation of variants. The best performance was defined as the method with the highest accuracy in the recognition of pathogenicity in both positive and negative datasets. On this benchmark, vERnet-B outperformed all other methods in Figure 3D at predicting clinical pathogenicity for missense SNVs in the BRCT domain of BRCA1. We drew ROC and PR curves to further compare the performance of vERnet-B with other methods (Figure 3E). It was found that vERnet-B offered a clear advantage over other methods in balancing the abilities of recognizing both the pathogenic and the benign variants.

### Combining vERnet-B with other evidence enhanced pathogenicity recognition

**Figure 4:**
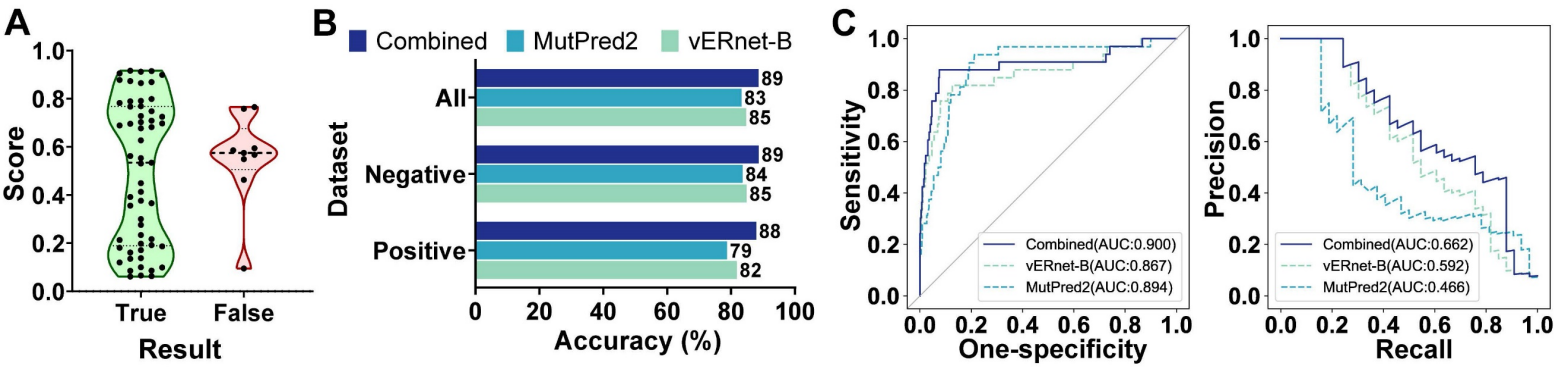
**|** Combination of vERnet-B with other evidence. (A) The MutPred2 scores with uncertain pathogenicity were around the threshold of 0.611(ranging from 0.45 to 0.772), thus the correct pathogenicity predictions mostly were scores outside this defined range. (B) Testing accuracy in the positive, negative, and integrated testing datasets for vERnet-B alone, MutPred2 alone, and vERnet-B combined with MutPred2. (C) The AUC values of the ROC and PR of the recognized pathogenicity ranks (bigger ranks refer to stronger risks) for the 484 BRCT variants in the testing dataset were shown. The solid curve represented the performance of the results combined with vERnet-B and MutPred2, while the dashed curves represented the performance of respectively using the vERnet-B and MutPred2 alone.

vERnet-B provided a single source of evidence that is well suited to be combined with other orthogonal sources of evidence (for example, the computational predictors mentioned for performance comparisons) for enhancing the pathogenicity recognition capacity. MutPred2 was also developed by machine learning with an output of the pathogenicity score ranging from 0 and 1, in which the higher score indicates the greater possibility of pathogenicity 16. MutPred2 set a common threshold of 0.611 for pathogenicity score to classify the pathogenicity. If the pathogenicity score of a variant was around 0.611, the strength of evidence in terms of pathogenicity was weak. In another word, this variant was unable to be determined as pathogenic nor benign, and thus it should be classified as VUS. As shown in Figure 4A, the pathogenicity scores of MutPred2 output ranging from 0.45 to 0.772 were defined as proximal to threshold 0.611, and these variants usually have contradictory pathogenicity recognition results from MutPred2 compared to ClinVar annotation and experimental assay. Therefore, we used vERnet-B as a supplement to MutPred2 to recognize these variants with scores proximal to threshold 0.611, which gained the best performance compared to MutPred2 or vERnet-B alone (Figure 4B). In the testing dataset, the recognition accuracy of MutPred2 alone, vERnet-B alone, and vERnet-B combined with MutPred2 was 83%, 85%, and 89%, respectively. The true negative detection rate of MutPred2 alone, vERnet-B alone, and vERnet-B combined with MutPred2 was 84%, 85%, and 89%, respectively. The true positive detection rate of MutPred2 alone, vERnet-B alone, and vERnet-B combined with MutPred2 was 79%, 82%, and 88%, respectively. The improvement in the AUC of the ROC and PR when using the combined results were recognized in the whole testing dataset (Figure 4C).

### Contribution of each network feature to model performance

**Figure 5:**
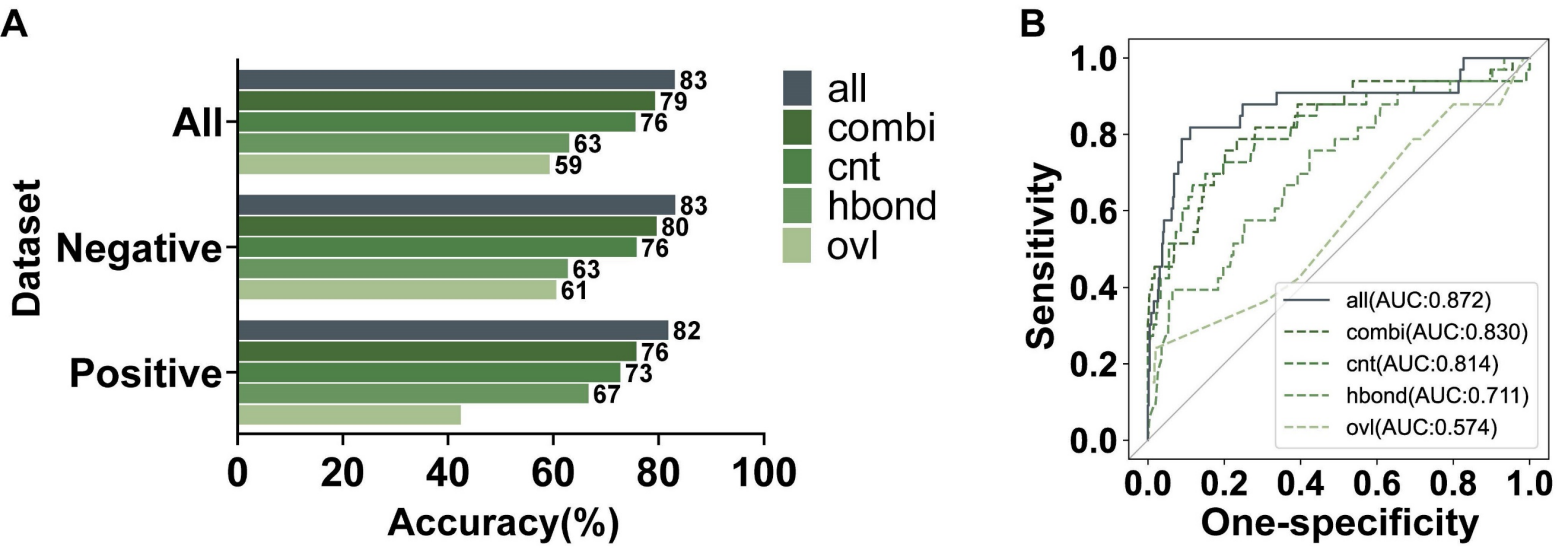
**|** Evaluation on the contribution of features in AANs. (A) Testing accuracy in the positive, negative, and integrated testing datasets by models using all features or an individual feature. (B) The AUC values of the ROC of the recognized pathogenicity ranks (bigger ranks refer to stronger risks) for the 484 BRCT variants in the testing dataset were shown. The solid curve represented the performance of the model using all features jointly, while the dashed curves represented the performance of the models using an individual network feature.

We further investigated the effectiveness of various features in ANNs for our model. There are three features with a single type of interaction, including hydrogen bonds (hbond), overlaps (ovl), and interatomic contacts (cnt). The weighted combination of these single features was added into the networks as a new feature, generic residue interaction (combi). Each feature was individually used to train a model, which was also evaluated by the testing dataset with 484 variants. As shown in Figure 5A, all the features were valuable for our recognition model to different degrees. To compare the contribution of each feature intuitively, we calculated the AUC values of ROC (Figure 5B). Above these features, the information about interatomic contacts had the most significant effect on recognizing pathogenicity. The interatomic contacts consist of some secondary bonds other than hydrogen bonds, in which the hydrophobic interactions are the dominant driving force for maintaining protein tertiary structures 33. Therefore, interatomic contacts are highly important for protein stability and function. Figure 2F also indicated that the channels of cnt involved the most abundant changes. The contribution of overlaps to our model was significantly minor, which was consistent with the conclusion in the previous research that only slight overlaps were marginally favorable 34. Furthermore, the combined feature had better performance than any single feature. However, since adding the single features can furtherly improve the performance of the learning model, we used both the weighted combined feature and the single features to jointly train our model.

### Pathogenicity recognition for other variants

**Figure 6:**
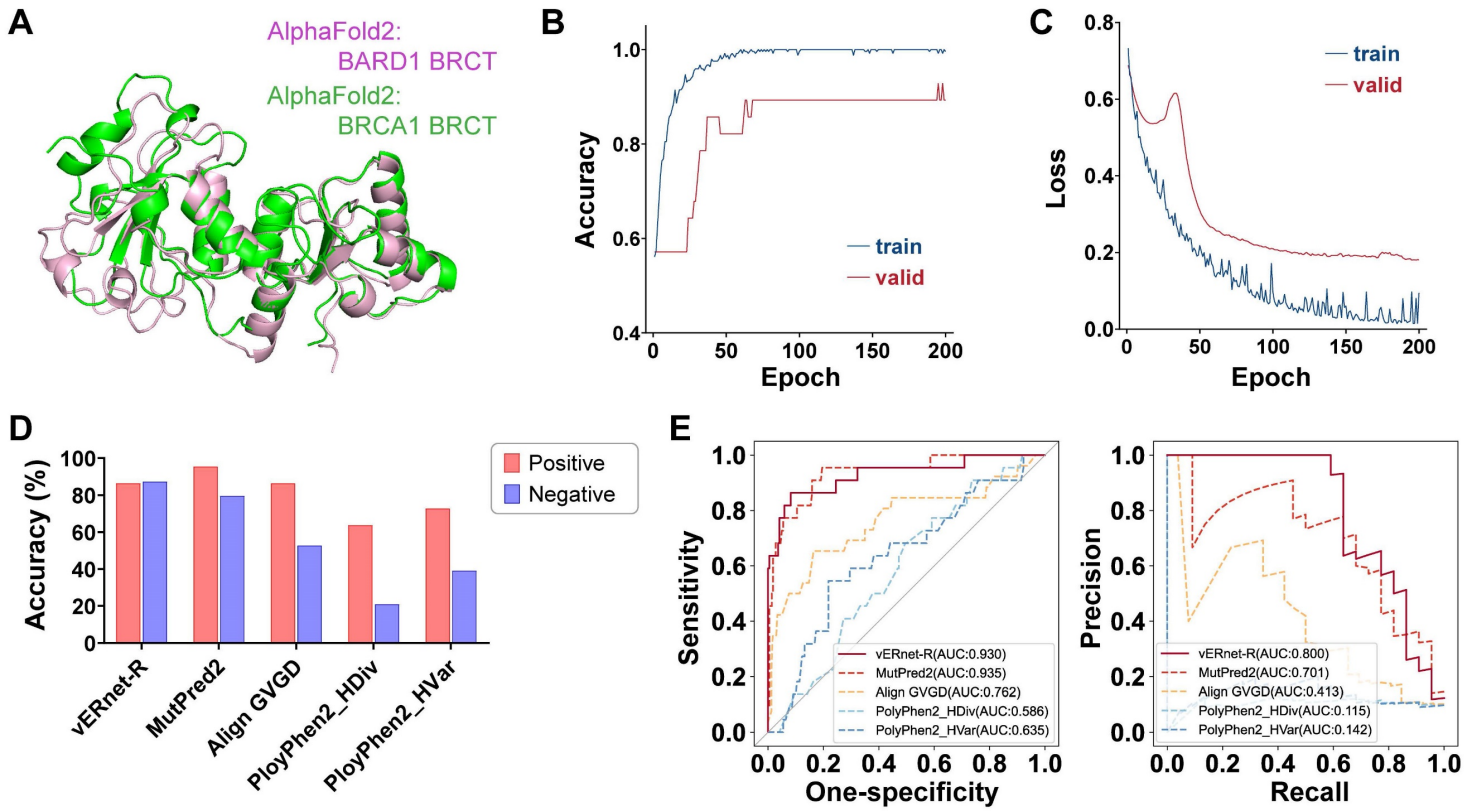
**|** Generalization on other genes. (A) Alignment and overlay of AlphaFold2-predicted BRCT domains of BRCA1 (green) and BARD1 (pink), with an average Cα r.m.s.d. of 1.639 Å. (B) The accuracy validation of vERnet-R over training time, in units of epochs. The curves were differentiated by colors to represented the accuracy on the training cohort (blue) and the independent validation cohort (red). (C) The loss function validation of vERnet-R over training time, in units of epochs. The curves were differentiated by colors to represented the loss function on the training cohort (blue) and the independent validation cohort (red). (D) Performance comparison of vERnet-R to other four computational variant effect predictors in the positive (red) and the negative (blue) testing datasets. (E) The AUC values of the ROC and PR of the recognized pathogenicity ranks (bigger ranks refer to stronger risks) for the 242 RING variants in the testing dataset were shown. The solid curve represented the performance of vERnet-R, while the dashed curves represented the performance of other four computational variant effect predictors.

vERnet-B is unable to be directly applied to other genes for pathogenicity recognition. In order to precisely identify the effect of small structure changes caused by one amino acid substitution, our method required that an individual recognition model should be trained for each protein. For example, vERnet-B failed in pathogenicity recognition for the BRCT domain of BARD1. BARD1 has homology BRCT domain of BRCA1, and the stable interaction between BRCA1 and BARD1 is essential for BRCA1 as a tumor suppression 35. It is equally important to infer the pathogenicity of BARD1 mutations. Although the BRCT domain is highly conserved, the average Cα r.m.s.d. between the BRCT of BRCA1 and BARD1 was 1.639 Å (Figure 6A), which was much higher compared to the average Cα r.m.s.d. between missense mutant BRCT and WT BRCT of BRCA1. Therefore vERnet-B mistakenly recognized all BRCT variants of BARD1 as pathogenic variants.

To validate our method and demonstrate its value as a discovery tool of variant interpretation, we applied this schema to the RING domain of BRCA1, which was a fragment containing 104 amino acids independent of the BRCT. We collected 521 missense SNVs in RING from the aforementioned platforms, in which the 279 variants were used to train a model for recognizing the pathogenicity of RING variants (see Supplementary Information). This model, named vERnet-R, was independent of vERnet-B and was trained by the same procedure as vERnet-B. As shown in Figure 6B-C, the accuracy and loss function on both the training and the validation datasets converged well within 200 training epochs. The remaining 242 RING SNVs were used for evaluating the performance of vERnet-R. vERnet-R achieved an accuracy of approximately 87% for classifying the pathogenic and benign RING variants, with a sensitivity of 86% and a specificity of 87%. Four computational variant effect predictors, with relatively good performance in the above work, were selected to be compared with vERnet-R. vERnet-R outperformed all other methods in our testing dataset (Figure 6D-E). The successful application on the RING domain proved the generalizable feasibility of our approach on other genes.

## Methods

### Construction of amino acid networks

Construction of AANs for protein variants in the current datasets (1143 variants) was performed to train a deep-learning model vERnet-B or evaluate its performance. As some successful practices of using AANs to infer protein functions have been achieved in previous studies 36-38, the atomic 3D coordinates of a protein used in the process of construction were predicted by AlphaFold2 18, 19, and processed using the Probe progress, version 2011.10 (http://kinemage.biochem.duke.edu) 39. In brief, the Probe identified contacts between amino acids in a protein by using a small rolling probe to evaluate their atomic packing. The program created a small virtual probe sphere (usually 0.25Å radius) that rolled around the van der Waals surface of each atom. If this probe touched or overlapped with another non-covalently bonded atom, it indicated that an interaction or an overlap was detected, which was represented by periodically drawing contact dots or spikes. The strength of interactions also could be quantitatively measured by the contact dots or spikes. Overlaps like hydrogen bonds were quantified by the volume of overlap, and other non-overlapping contacts were quantified by a weighted sum of the contact scores per dot. The combined score for generic residue interaction was obtained by summing the weighted scores of these three interactions. The scores of interactions were proportional to their strength. Next, the Probe program summarized and outputted these scoring data for all atoms of an entire structure to a file. The final step to generate networks was completed by the Python package RINerator, version 2014.10 (https://rinalyzer.de/rinerator.php) 24. It integrated the information for every atom or residue from the previous file to construct an undirected weighted network with multiple edges, in which the nodes and edges respectively represented the amino acid residues and the non-covalent interactions. There are four possible types of edge, including interatomic contact (cnt), overlap (ovl), hydrogen bond (hbond), and generic residue interaction (combi), while the subtypes of each interaction are combinations between main chains (mc) and side chains (sc). The resulting AANs were stored in files formatted as SIF (non-weighted) and NA (weighted).

### Data preparation for the learning of AANs

Six hundred and fifty-nine BRCA1 variants were selected and downloaded from three platforms, including ClinVar (https://www.ncbi.nlm.nih.gov/clinvar) 7, BRCAExchange (https://brcaexchange.org) 23, and SGE function scores (https://sge.gs.washington.edu/BRCA1) 10, to train the 2D-CNN model. They were all missense SNVs that only caused the substitution of one amino acid in the BRCT domain. To evaluate the potential of vERnet-B for inferring variant pathogenicity, four hundred and eighty-four variants of the same type were selected and downloaded from these platforms, the amino acid substitutions caused by which were different and not present in the training dataset. To prepare structure information of protein variants as input data for vERnet-B, we carried out the following data preparation. Initially, the sequences of BRCT fragments were generated using the HGVS format representations of variants for the tertiary structure prediction with AlphaFold2. Subsequently, the files saving the AANs were generated using the method in the previous section. The resulting AANs were transformed into 3D matrices, where the coordinates of both the row and column represented the amino acids in a sequence and the element values represented the interaction strengths between amino acids at the corresponding positions. Here, the shape of the input data is 214×214 grid with 7 channels. For each interaction type, the interactions between main-chain to main-chain and side-chain to side-chain were aggregated into one channel, while the interactions between main-chain to side-chain were stored in a separate channel. All preprocessing procedures were performed using Python.

### Construction of deep neural networks

The architecture of the learning model used to implement the base classifiers of vERnet-B was ResNet18 31, including 2D convolutional blocks and fully connected blocks. The 2D convolutional blocks consisted of one max-pooling layer and five 2D convolutional layers. The first 2D convolutional layer (with the kernel size of 7×7) was applied before max-pooling. Residual blocks were implemented to form the other four 2D convolutional layers, each containing parallel convolutions. At the end of each convolution operation, batch normalization (BN) and Leaky ReLU activation were adopted 40, 41. Each of the last four 2D convolutional layers stacked two residual blocks, and different channel sizes (64, 128, 256, and 512) were applied to them, with the kernel size of 3×3. Down-sampling was used in the first 2D convolutional layer and the first residual block of the last three 2D convolutional layers. The maximum pooling size was set to 3×3. Fully connected blocks consisted of the global average pooling layer and dense layer. The cross-entropy was used as the loss function. A total of 11,194,882 trainable parameters were included in the model. An overview of the deep neural networks was provided in Figure S1. The number of epochs and batch size hyperparameters were set to 200 and 26, respectively, and we used early stopping with a patience interval of ten epochs in order to prevent overfitting. The learning rate was automatically chosen by Keras's optimizer, Adadelta 42. To evaluate the training effect of our 2D-CNN model, we assigned 10% of the training samples to the validation dataset and the remaining 90% to the training dataset. We trained 3 models with good performance to ensemble vERnet-B, all of which were trained using an NVIDIA Corporation GP102 with 32GB of memory. The Keras library 2.5.0 with TensorFlow 2.5.0 as backend was used for the implementation of the 2D-CNN model 43, 44.

### EasyEnsemble

One of the popular methods in dealing with class-imbalance problems is under-sampling. The traditional under-sampling methods only used a subset of the majority class 45, so it's not efficient in the usage of the samples. EasyEnsemble was proposed to overcome this deficiency by using a set of majority class examples N and a set of minority class examples P 30. Specifically, we firstly randomly sampled a subset Ni from N with the same number as P. Then T base classifiers were trained using different under-sampled datasets, of which the outputs were integrated together as the final model result. Here, we did not use the sgn function to obtain the ensemble results but chose a more reliable probability to decide the classification.

## Discussion

This work succeeded in the recognition of the pathogenicity of missense SNVs in BRCA1. vERnet-B considerably outperformed other computational methods in terms of accurate and unbiased recognition of variant pathogenicity, and combining vERnet-B with other evidence further enhanced the model performance. vERnet-B has the potential to facilitate the clinical application of genetic information by interpreting VUS. For example, determining the appropriate classification of VUS can aid genetic counseling 46, because deleterious BRCA1 variants are likely to increase cancer risk. In addition, reliable VUS classification can also guide the cancer precision treatment, as hypersensitive to PARP inhibitors (Olaparib) was observed in tumors with loss-of-function BRCA1 mutations 47.

The fact that the majority of variants in ClinVar still had uncertain or conflicting significance illustrated the challenges posed by VUS. The traditional approaches for interpreting VUS were experimental assessments 8-10, which were limited mainly by their high cost. Previous studies have developed AI-based variant effect predictors using primary sequences 13-16, which demonstrated that AI could accelerate the interpretation of variants at a negligible cost. Our work furtherly directed a novel path that the protein tertiary structures predicted by AlphaFold2 could be used to efficiently extract features associated with variant pathogenicity using the deep-learning technique. However, some authors reported that AlphaFold2 had a defect in predicting the impact of missense mutations 22. We have overcome it by constructing the AANs from AlphaFold2-predicted structures to deduce more biochemical information. Take BRCT-A1708E as an example, the impact of this mutation seems to be poorly predicted by AlphaFold2 in the prevailing view, but our method successfully recognized it as a pathogenic mutation through its structure. Therefore, a sufficient number of protein tertiary structures could be obtained for deep learning.

The potential problem caused by the inconsistent interpretation of several variants in our raw dataset was a caveat of the current research. Although we had deleted some of these ambiguous data, some unfaithful samples with single evidence might still be included. We have carried out a simple solution, cross-training, to eliminate the influence of such unfaithful samples. We pre-trained a CNN model using some independent samples to recognize the training dataset. Two samples that were incorrectly recognized with extreme scores, which indicated they were difficult samples or misclassified samples, were removed from the training dataset. Optimized data screening methods are expected in future studies.

The major limitation of this method was the inevitable conflict between precision and generalization ability. The current method required training an individual recognition model for each protein. While the vERnet-B cannot be directly generalized to other genes, we had a successful application on RING domain of BRCA1 by training an individual model for it. It is conceivable that further investigation on this method will make it more useful by getting a better balance between precision and generalization.

AlphaFold2 provides an optimal static structure, while the state of compound binding may cause conformational changes in proteins. However, although the molecular dynamics simulation is theoretically appealing, this approach is highly challenging for even moderate-sized proteins due to the complexity and context dependency of protein stability 18. Our data showed that the static structures provided by AlphaFold2 were reliable for extracting pathogenicity-related features of variants. It is expected that molecular dynamics simulation coupled with AlphaFold2 may further optimize our model.

In conclusion, we proved that the relationship between AlphaFold2-predicted protein tertiary structures and the pathogenicity of missense SNVs is learnable. The construction of AANs is a critical step for applying protein tertiary structures to deep learning. This method paved the way for generating variant effect learning models with even higher accuracy in the future. vERnet-B will spur clinical genetic information research and enrich the toolbox of design and implementation of precision treatment.

## Supplementary Material

Supplementary figure and tables.

## Abbreviations

AAN: amino acid interaction network
AI: artificial intelligence
AUC: area under the curve
CNN: convolutional neural networks
Cα: α-carbon
r.m.s.d.: root-mean-square deviation
PDB: protein data bank
pLDDT: predicted local-distance difference test
PR: precision-recall
ROC: receiver operating characteristic
SGE: saturation genome editing
SNV: single-nucleotide variant
vERnet-B: variant effect recognition network for BRCA1
VUS: variant of uncertain significance
WT: wild-type
