# Supplementary material for: Artificial intelligence-based recognition for variant pathogenicity of BRCA1 using AlphaFold2-predicted structures
Source: Theranostics. 2023 Jan 1;13(1):391–402. doi: 10.7150/thno.79362 (PMC9800725; doi:10.7150/thno.79362)
Supplement: Supplementary file 1 — Supplementary figure and tables. [file thnov13p0391s1.pdf]

Figure S1 | Overview of the 2D-CNN model of PSFnet. The model was implemented by ResNet18 and was composed of two types of blocks, i.e., 2D convolutional blocks, and fully connected blocks. The 2D convolutional blocks consisted of one max-pooling layer and five 2D convolutional layers, containing two residual blocks in each 2D convolutional layer. The first 2D convolutional layer was applied before max-pooling with the kernel size of 7×7. Each residual block had two convolutions and was end up with batch normalization and Leaky ReLU activation. The kernel size for convolution in residual blocks and the maximum polling size were set to 3×3 and 3×3, respectively. Each of the last four 2D convolutional layers adopted different channel sizes (64, 128, 256, and 512). Down-sampling was used in the first 2D convolutional layer and the first residual block of the last three 2D convolutional layers. Fully connected blocks consisted of the global average pooling layer and dense layer. The cross-entropy was used as the loss function. A total of 11,194,882 trainable parameters were included in the model.

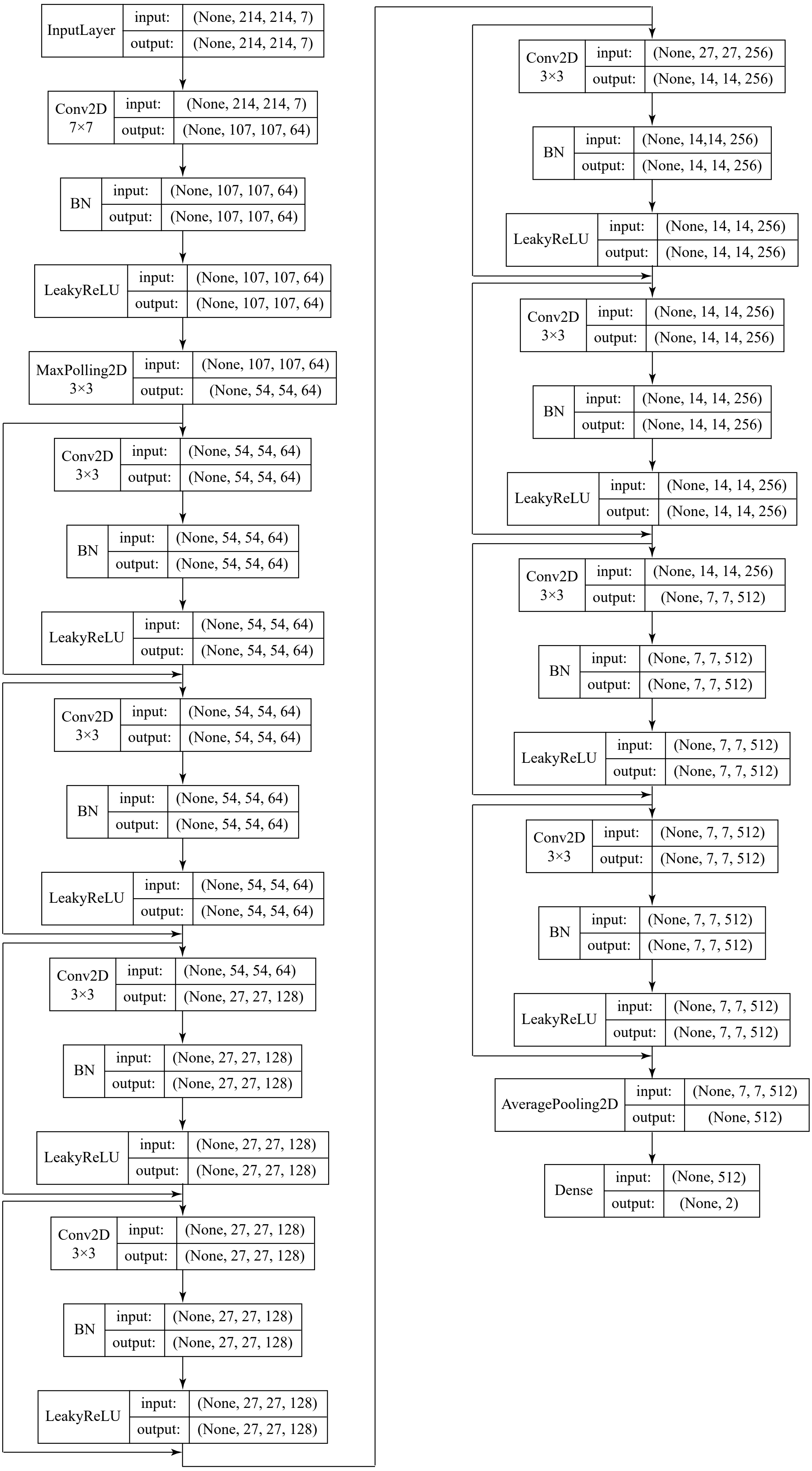

Table S1 | List of amino acid substitution for 659 variants used in the vERnet-B training.

CLASS #	Protein variants
**Positive**	R1753S, R1753S, R1753I, R1753T, R1753K, R1753G, A1752E, A1752V, A1752T, A1752P, R1751P, R1751G, P1749Q, P1749L, P1749S, G1748D, G1748R, G1748C, Q1747P, H1746P, H1746D, H1746Y, Q1848P, G1743V, G1743E, G1743R, G1743R, V1741D, D1739E, D1739E, D1739A, D1739V, D1739G, D1739H, D1739Y, G1738A, G1738E, V1736D, V1736G, V1736F, V1736L, E1735D, E1735G, E1735K, F1734L, F1734L, F1734C, F1734S, F1734V, F1734I, F1734L, E1731K, Q1846P, S1722P, Q1721P, V1719E, V1719G, W1718C, W1718L, W1718R, W1718R, W1718G, S1715R, S1715I, S1715C, V1714D, V1714G, V1714F, V1713G, V1713E, V1713A, V1713L, W1712G, K1711E, G1709V, A1708G, A1708P, A1708T, I1707S, I1707F, G1706V, G1706R, L1705P, F1704L, F1704L, F1704C, F1704S, F1704Y, F1704V, F1704I, F1704L, Y1703S, Y1703C, Y1703N, Y1703D, Y1703H, K1702N, K1702N, K1702I, K1702R, K1702T, K1702E, K1702Q, T1700K, T1700I, T1700R, T1700P, T1700A, R1699L, R1699P, R1699G, C1697W, C1697F, C1697Y, V1696E, E1694K, A1693D, A1693V, A1693S, D1692E, D1692E, D1692A, D1692V, D1692N, A1843E, T1691R, T1691I, T1691K, T1691P, M1689K, M1689R, A1843P, V1688D, V1688G, V1688F, V1687D, V1687G, V1687F, H1686Q, H1686Q, H1686L, H1686P, H1686D, H1686N, H1686Y, T1685S, T1685N, T1685P, V1842G, T1684I, T1684P, V1842A, E1683Q, E1683K, V1842E, E1682V, T1681P, S1841R, S1841R, S1841I, S1841T, S1841N, H1672P, R1670K, A1669P, S1841R, F1668L, F1668S, D1840E, V1665E, D1840V, D1840G, M1663R, M1663K, M1663L, L1657R, L1657Q, L1657P, S1655C, S1655Y, S1655F, S1655P, V1653E, V1653L, V1653L, M1652R, M1652K, L1839V, S1651P, V1838G, W1837C, W1837C, W1837L, W1837S, W1837G, E1836V, R1835G, V1833E, V1833M, V1832G, I1824M, A1823S, A1823P, D1818G, D1818H, W1815C, W1815C, W1815S, A1814G, P1812A, Q1811L, Q1811P, Q1811R, Q1811K, V1810E, V1810G, V1809D, V1809A, V1808G, I1807S, I1807N, Y1853S, Y1853C, Y1853D, Y1853H, Y1853N, A1789D, A1789V, A1789P, A1789T, G1788A, G1788R, G1788S, G1788C, C1787W, C1787Y, C1787R, L1786P, Q1785P, V1784E, M1783R, M1783K, L1780R, L1780P, M1775V, G1770W, G1770R, Y1769D, C1768W, C1768F, I1766T, L1764Q, G1763V, F1761C, F1761S, F1761V, F1761I, I1760S, I1760T, I1760N
**Negative**	R1758K, R1758G, D1757E, E1849D, D1757A, E1849D, Q1756L, Q1756R, Q1756E, S1755C, S1755F, E1754D, E1754A, E1754Q, A1752G, A1752S, R1751L, E1849Q, K1750M, K1750R, K1750Q, P1749T, P1749A, G1748A, G1748S, Q1747H, Q1747L, Q1747R, H1746L, H1746R, N1745I, N1745H, R1744S, R1744K, R1744G, N1742K, N1742T, N1742S, V1741A, V1741I, V1740G, V1740A, V1740L, D1739N, R1737I, R1737T, V1736I, E1735V, E1735A, E1735Q, F1734Y, C1847G, D1733E, C1847R, H1732L, H1732P, H1732R, E1731D, E1731A, N1730Y, L1729Q, L1729V, M1728T, M1728K, Q1846L, M1728L, K1727T, Q1846R, K1727E, K1727Q, R1726G, E1725D, E1725G, K1724R, K1724E, I1723T, I1723F, S1722C, S1722A, Q1721H, T1720S, V1719A, V1719L, Y1845F, F1717L, F1717C, Y1716F, Y1716S, Y1716H, S1715T,

CLASS #	Protein variants
	Y1845C, V1714L, V1714I, V1713I, W1712C, W1712C, W1712R, K1711N, Y1845H, K1711R, G1710R, G1710R, G1709A, G1709R, G1709R, A1708V, A1708S, I1707T, I1707V, I1707L, L1705Q, L1705I, L1705V, Y1703F, L1701V, L1701M, E1698D, E1698D, C1697S, C1697S, V1696A, V1696M, V1696L, F1695L, F1695L, E1694D, E1694A, A1843G, A1693P, A1693T, T1691S, K1690N, A1843S, K1690R, A1843T, M1689T, M1689L, M1689V, V1688A, V1688I, V1687L, V1687I, T1685S, T1684S, T1684A, E1683D, E1683D, E1683A, E1682G, E1682A, T1681N, T1681S, V1842L, T1681A, I1680T, V1842I, I1680V, I1680L, L1679V, N1678D, T1677S, T1677S, L1676F, T1675P, T1675S, T1675A, I1674V, I1674L, H1673L, H1673R, H1673Y, H1672L, S1841C, K1671R, K1671T, R1670S, R1670G, A1669G, A1669T, F1668L, F1668Y, F1668L, K1667N, K1667Q, K1667E, Y1666C, Y1666N, Y1666H, V1665A, V1665L, V1665M, L1664H, L1664F, L1664I, M1663I, M1663I, M1663V, D1840A, F1662C, F1662I, E1661D, E1661Q, E1660D, P1659Q, D1840N, P1659T, T1658S, L1657V, L1839F, G1656A, G1656C, L1839F, S1655T, V1654A, V1654L, V1653G, V1653A, M1652L, S1651A, S1651T, M1650R, M1650V, R1649I, R1649G, K1648E, V1838A, N1647S, N1647T, N1647Y, V1646A, V1646D, V1646G, V1838L, V1838L, E1836D, E1836A, T1834A, T1834P, V1833A, V1832L, P1831L, P1831A, A1830V, A1830S, A1830T, E1829D, E1829V, C1828W, C1828S, M1827T, M1827L, Q1826R, Q1826E, G1825A, G1825E, G1825R, I1824S, I1824N, A1823V, H1822Q, H1822D, H1822N, F1821Y, F1821L, G1820V, G1820R, N1819K, N1819S, N1819D, D1818E, I1855L, E1817G, T1816I, T1816R, W1815L, W1815R, W1815R, A1814V, A1814S, A1814T, P1812R, P1812T, Q1811H, Q1811H, Q1811E, V1810A, V1810L, L1854R, V1809I, V1808L, I1807M, L1854Q, I1807L, I1807V, P1806S, P1806A, H1805L, H1805Y, L1854V, V1804I, V1804F, G1803R, G1803C, T1802I, T1802P, G1801V, G1801S, T1799S, T1799A, F1798S, S1797A, S1797P, Y1853F, S1796A, S1796P, S1796T, L1795R, L1795F, E1794G, E1794A, K1793R, K1793T, K1793Q, V1792A, V1792M, V1791A, V1791L, V1791L, S1790C, S1790A, A1789G, A1789S, G1788D, C1787F, C1787G, L1786R, L1786V, L1786M, Q1785H, T1852N, V1784A, V1784I, M1783I, M1783L, W1782S, W1782R, E1781A, E1781Q, T1852P, L1780V, Q1779H, Q1779K, T1852A, D1778E, D1778A, D1851E, T1777A, T1777S, P1776R, P1776H, M1775I, M1775L, N1774T, N1774D, N1774H, T1773I, T1773S, F1772S, F1772Y, F1772L, P1771S, G1770E, D1851H, G1770A, Y1769F, Y1769N, C1768S, C1768Y, C1767F, C1767Y, I1766L, I1766V, E1765D, E1765K, L1764V, L1764I, G1763A, G1763R, R1762M, R1762W, F1761L, F1761L, F1761L, I1760M, I1760V, I1760L, K1759M, L1850M, R1758S

Table S2 | List of amino acid substitution for 484 variants used to evaluate the vERnet-B.

CLASS #	Protein variants
**Positive**	P1749R, G1738V, G1738R, V1736A, F1734I, S1722F, S1715N, A1708E, G1706E, R1699Q, R1699W, C1697R, V1696G, F1695V, D1692Y, D1692H, H1686R, T1685I, T1685A, E1683V, L1839S, V1838E, W1837R, A1823T, L1854P, Y1853S, G1788V, M1775K, M1775R, G1770V, I1766N, I1766S, L1764P
**Negative**	R1758I, R1758T, D1757V, D1757G, D1757N, D1757Y, D1757H, Q1756H, Q1756P, Q1756K, S1755Y, S1755A, S1755P, S1755T, E1754V, E1754G, E1754K, E1849G, R1751Q, K1750N, K1750T, E1849K, K1750E, Q1848H, Q1747E, Q1747K, Q1848L, Q1848R, N1745K, N1745T, N1745S, N1745Y, N1745D, R1744I, R1744T, Q1848E, Q1848K, N1742I, N1742H, C1847W, R1737S, C1847S, R1737G, C1847Y, C1847F, D1733A, D1733V, D1733G, D1733Y, D1733H, D1733N, H1732Q, H1732Y, H1732D, H1732N, E1731V, E1731G, Q1846H, N1730K, N1730T, N1730I, N1730S, P1859R, N1730D, N1730H, L1729R, L1729M, M1728I, M1728R, M1728V, K1727N, K1727I, K1727R, R1726S, R1726K, R1726T, R1726I, E1725V, Q1846E, E1725A, E1725Q, E1725K, K1724N, K1724I, Q1846K, K1724T, K1724Q, I1723M, I1723S, I1723N, I1723L, I1723V, S1722T, Q1721L, Q1721R, Q1721K, Q1721E, T1720A, V1719M, F1717S, Y1845S, F1717Y, F1717V, F1717I, Y1716C, Y1716D, Y1716N, W1712L, W1712S, K1711I, K1711T, K1711Q, Y1845N, G1710V, G1710A, G1710E, G1709E, I1707M, I1707N, G1706A, L1844H, L1844P, L1701R, L1844R, L1701Q, L1844F, L1844V, E1698V, E1698G, E1698A, E1698Q, L1844I, E1698K, C1697G, F1695C, F1695S, F1695Y, E1694V, E1694G, E1694Q, A1843V, K1690E, K1690Q, M1689I, V1688L, V1687A, E1682D, I1680M, I1680S, I1680N, I1680F, L1679R, L1679Q, L1679P, L1679I, N1678K, N1678I, N1678S, N1678T, N1678Y, N1678H, T1677N, T1677A, T1677P, L1676V, L1676I, T1675N, T1675I, I1674T, H1673Q, H1673P, H1673N, H1673D, H1672Q, H1672R, H1672N, H1672Y, K1671N, K1671I, K1671E, K1671Q, R1670I, A1669S, F1668C, F1668V, F1668I, K1667M, K1667T, K1667R, Y1666F, Y1666S, Y1666D, V1665G, L1664R, L1664P, L1664V, F1662L, F1662Y, F1662S, F1662V, D1840H, E1661V, E1661A, E1661K, E1660V, E1660G, E1660A, E1660Q, E1660K, P1659L, P1659R, P1659S, P1659A, T1658A, L1657M, G1656V, G1656D, G1656R, G1656S, V1654G, V1654M, M1652I, M1652T, M1652V, S1651C, S1651Y, S1651F, M1650I, M1650K, M1650T, M1650L, R1649S, R1649K, R1649T, K1648N, K1648I, K1648R, K1648T, K1648Q, N1647K, N1647I, N1647D, N1647H, V1646L, V1838M, I1858L, E1836G, E1836Q, E1836K, T1834S, T1834N, T1834I, P1831H, P1831R, P1831S, P1831T, A1830P, E1829G, E1829A, E1829Q, P1856S, C1828F, C1828Y, C1828G, M1827I, M1827R, M1827K, I1855M, M1827V, Q1826H, Q1826P, Q1826L, Q1826K, G1825V, G1825W, I1855R, I1824T, I1824F, I1824L, I1824V, A1823E, H1822L, H1822R, H1822P, H1822Y, I1855T, F1821C, F1821S, F1821V, F1821I, G1820D, G1820C, G1820S, N1819I, N1819T, N1819Y, N1819H, I1855V, D1818V, D1818A, D1818Y, D1818N, E1817D, E1817A, E1817V, E1817Q, E1817K, T1816K, T1816S, T1816A, T1816P, W1815G, D1813E, D1813G, D1813A, D1813V, D1813N, P1812S, V1810M, I1807T, P1806L, P1806R, P1806Q, P1806T,

CLASS #	Protein variants
	H1805Q, H1805R, H1805P, H1805D, H1805N, V1804G, V1804A, V1804D, V1804L, L1854M, G1803V, G1803A, G1803S, T1802R, T1802K, T1802S, T1802A, G1801A, G1801D, G1801C, G1801R, L1800P, L1800H, L1800R, L1800V, L1800I, L1800F, T1799N, T1799I, T1799P, F1798L, F1798C, F1798Y, F1798V, F1798I, S1797L, S1797T, L1795P, L1795H, L1795V, L1795I, E1794D, E1794V, E1794Q, E1794K, K1793N, K1793M, K1793E, V1792G, V1792L, V1791G, V1791M, S1790F, S1790Y, S1790T, S1790P, C1787S, L1786Q, T1852S, Q1785L, Q1785R, Q1785E, Q1785K, V1784G, V1784L, M1783T, M1783V, W1782C, T1852I, W1782L, W1782G, E1781D, E1781V, E1781K, Q1779L, Q1779R, Q1779P, Q1779E, D1778V, D1778G, T1777R, T1777K, T1777I, T1777P, P1776L, P1776A, P1776T, P1776S, M1775T, N1774K, N1774I, N1774S, D1851V, N1774Y, T1773N, D1851G, T1773P, F1772C, D1851A, F1772I, P1771H, P1771L, P1771R, P1771A, D1851Y, P1771T, Y1769S, D1851N, C1767S, I1766M, I1766F, E1765V, E1765A, E1765Q, L1850R, L1850P, R1762S, R1762T, R1762K, L1850Q, R1762G, L1850V, K1759N, K1759T, K1759R, K1759E, K1759Q

Table S3 | List of amino acid substitution for 279 variants used in the vERnet-R training.

CLASS #	Protein variants
**Positive**	G98V, T97P, D96E, D96V, D96A, D96G, D96Y, D96H, D96N, L95P, Q94L, F93L, F93S, A92V, A92D, A92T, A92P, I89N, L86R, L86P, E85G, E85K, E84V, E84K, V83D, V83F, L82R, L82P, L82H, L82F, Q81H, S80I, L73R, L73Q, S72N, R71S, R71M, R71W, I68R, I68K, N66Y, C64W, C64F, C64S, C61W, C61F, C61S, C61Y, P58H, L51P, L49P, M48R, M48K, C47W, C47S, C47F, C47R, C47G, K45I, C44W, C44S, C44G, F43L, H41Q, H41P, H41L, H41D, H41Y, H41N, C39S, C39F, C39G, T37R, V35G, V35A, V35D, P34R, P34H, P34L, P34S, L28P, C27W, C27Y, C27F, C27G, C27R, C27S, I26T, P25L, C24W, C24S, C24Y, C24G, C24R, Q19P, M18R, M18K, A17P, I15S, I15N, I15T, V14G, V14F, Q12P, V11E, V11G, D2H, M1I, M1T, M1L
**Negative**	Y101D, Y101N, E100D, L99V, G98S, G98R, T97S, T97A, L95R, L95I, Q94R, Q94E, F93Y, F93V, A92S, C91Y, C91S, I90T, I90V, I89T, I89L, K88N, K88E, L87F, L87S, L86Q, L86I, E85V, E85Q, E84G, E84Q, V83G, V83I, L82I, Q81R, Q81K, S80T, S80G, F79L, F79Y, R78S, R78T, T77S, T77A, S76C, S76G, E75D, E75A, Q74H, L73P, L73V, S72T, S72G, K70N, K70R, T69N, T69S, I68M, I68V, D67E, D67N, N66K, N66I, K65M, K65Q, L63V, L63I, P62L, P62A, Q60P, Q60R, S59A, S59T, P58L, P58R, G57V, G57A, K56I, K56Q, K55T, K55Q, Q54H, Q54R, N53S, N53H, L52V, L52F, L51H, L51F, K50R, K50T, L49Q, L49M, M48I, F46L, F46S, K45N, K45R, F43Y, I42M, I42V, D40E, D40G, K38T, K38Q, T37S, T37A, S36A, S36T, V35I, P34A, E33D, E33V, K32R, K32T, I31F, I31V, L30S, L30M, E29A, E29Q, L28R, L28Q, I26M, I26V, P25S, P25A, E23V, E23Q, L22F, L22V, I21S, I21L, K20I, K20R, Q19L, Q19R, M18L, A17D, A17S, N16I, N16T, I15V, I15L, V14A, V14I, N13T, N13D, Q12L, Q12R, V11A, V11I, E10D, E10A, E9D, E9A, V8G, V8A, R7L, R7C, L6P, L6V, A5D, A5V, S4A, S4T, L3V, L3I, D2E

Table S4 | List of amino acid substitution for 242 variants used to evaluate the vERnet-R.

CLASS #	Protein variants
**Positive**	C39R, C39W, C39Y, C44F, C44R, C44Y, C47Y, C61G, C64G, C64R, C64Y, H41R, I26N, L22S, M18T, M1K, M1R, M1V, R71G, R71K, R71T, T37K
**Negative**	A17G, A17T, A17V, A5G, A5S, A5T, C91F, C91G, C91R, D2A, D2N, D2Y, D40A, D40H, D40N, D40V, D40Y, D67A, D67G, D67H, D67V, D67Y, E10G, E10Q, E23A, E23K, E29D, E29G, E29K, E29V, E33A, E33K, E33Q, E75G, E75K, E84A, E84D, E85A, E85D, E9G, E9K, E9Q, E9V, F46C, F46I, F46V, F46Y, F79C, F79S, F79V, F93I, G57R, G98A, G98D, I15M, I21F, I21M, I21N, I21T, I21V, I31L, I31M, I31S, I31T, I42K, I42L, I42R, I42T, I68L, I68T, I89F, I89M, I90F, I90L, I90M, I90S, K20E, K20N, K20T, K32E, K32M, K32N, K32Q, K38E, K38M, K38N, K38R, K45E, K45Q, K45T, K50E, K50I, K50N, K50Q, K55E, K55M, K55N, K55R, K56E, K56N, K56R, K56T, K65E, K65N, K65R, K65T, K70E, K70I, K70Q, K70T, K88Q, K88R, K88T, L22I, L28M, L28V, L30F, L30V, L30W, L3F, L3S, L49R, L51I, L51R, L51V, L52I, L63F, L63S, L6F, L6H, L6I, L6R, L86V, L87M, L87V, L87W, L95F, L95H, M18I, M48L, M48T, M48V, N13H, N13I, N13K, N13S, N13Y, N16D, N16H, N16K, N16S, N16Y, N53D, N53I, N53K, N53Y, N66D, N66H, N66S, N66T, P25R, P25T, P58A, P58T, P62R, P62S, Q12E, Q12H, Q12K, Q19E, Q19H, Q19K, Q54E, Q54K, Q54L, Q54P, Q60E, Q60H, Q60K, Q60L, Q74E, Q74P, Q74R, Q81L, Q81P, Q94H, Q94K, R78G, R78I, R78K, R7G, R7H, R7P, S4C, S4F, S4P, S4Y, S59L, S59P, S72C, S72R, S76N, S76R, S76T, S80C, S80N, S80R, T37P, T69A, T69I, T69P, T77K, T77M, T77P, T77R, V14L, V83L, V8F, V8I, V8L
